# Relationship between Evaluations of Tracheal Tube Position Using Ultrasound and Fluoroscopy in an Infant and Pediatric Population

**DOI:** 10.3390/jcm9061707

**Published:** 2020-06-02

**Authors:** Davinder Ramsingh, Elizabeth Ghazal, Brent Gordon, Philip Ross, Darren Goltiao, Matt Alschuler, Justin Pugh, Matthew Holsclaw, Linda Mason

**Affiliations:** 1 Department of Anesthesiology, Loma Linda University Medical Center, 11234 Anderson Street, Loma Linda, CA MC-2532-D, USA; EGhazal@llu.edu (E.G.); pross@llu.edu (P.R.); dgoltiao@llu.edu (D.G.); malschuler@llu.edu (M.A.); jpugh@llu.edu (J.P.); mholsclaw@llu.edu (M.H.); lmason@llu.edu (L.M.); 2 Department of Pediatric Cardiology, Loma Linda University Medical Center, 11234 Anderson Street, Loma Linda, CA MC-2532-D, USA; BRGordon@llu.edu

**Keywords:** point-of-care ultrasound, pediatrics, airway ultrasound, tracheal tube positioning

## Abstract

Introduction: A non-radiographic technique to measure the location of the tracheal tube (TT) in children is of value given the risk of inappropriate TT placement along with concerns about radiation exposure. Airway point-of-care ultrasound (POCUS) has demonstrated utility in children, but the examinations vary by age and may require non-traditional techniques or utilize less common probes. This study evaluated the performance of measuring the tracheal location of the cuffed TT using a novel, linear probe-based POCUS examination over a wide age range of children. After adjusting for the subjects’ height and TT size, ultrasound measurements of the TT cuff location were compared with fluoroscopy measurements of the TT tip location. Methods: Perioperative pediatric patients (<10 years) requiring a cuffed TT were enrolled. After routine TT placement, ultrasound and fluoroscopy images were obtained. Measurements from the TT cuff to the cricoid cartilage were obtained from the POCUS examination. Chest fluoroscopy was reviewed to measure the TT’s distance from the carina. Both measurements were then compared after scaling for patient height. The duration of the ultrasound examination and image quality scores were also recorded. Results: Forty-one patients were enrolled, with a median age of 3 (25th/75th percentile: 1.50/7.00) years. The POCUS examination identified the TT cuff in all cases with the highest image quality score. The median POCUS exam time was 112 (25th/75th percentile: 80.00/156.00) seconds. There was a strong correlation between the POCUS measurements and the fluoroscopy measurements, r = −0.7575, 95% CI [−0.8638, −0.5866 ], *p* < 0.001). Conclusions: Our results demonstrate a strong correlation between POCUS TT localization measurements and traditional measurements via fluoroscopy. This study further supports the utility of POCUS for pediatric care.

## 1. Introduction

Tracheal intubation is a critical skill for acute care providers working with children. One of the most important aspects of tracheal intubation is the proper positioning of the tracheal tube (TT). Previous studies have demonstrated the incidence of TT malposition to be between 15% and 38% [1,2,3]. Additionally, several studies have demonstrated that a large percentage of malpositioned TTs are not recognized after auscultation and clinical examination [4,5]. A recent study by anesthesiologists demonstrated auscultation to be inaccurate when determining TT malposition in adults [6]. Verghese et al. demonstrated similar results in children, showing inappropriate TT positioning after the verification of bilateral breath sounds at a rate of 31% [5]. Other techniques used to evaluate appropriate TT placement include: (1) age/height-based formulas, (2) palpation of the trachea, (3) the evaluation of TT pilot balloon distention with sternal notch palpation, and (4) fluoroscopy/chest x-ray. However, each of these techniques has limitations [7,8].

Malposition of the TT can result in serious consequences such as: (1) hypoxemia, (2) inadequate ventilation, (3) pneumothorax, (4) barotrauma, (5) atelectasis, and (6) the potential for inappropriate interventions such as chest tube placement. In neonates and children, these concerns are magnified, due to the smaller margin of error when positioning the TT. Additionally, flexion and extension have shown to impact TT positioning in this population [9].

While not commonly used in the operating room, fluoroscopy has been used for confirmation of the correct position of the TT and has demonstrated benefits for the identification of TT malposition by allowing measurements of the placement of the TT within the trachea. [4]. However, disadvantages include: (1) the exposure to radiation, (2) the lack of immediate availability, and (3) the inability to perform frequent examinations.

Point-of-care ultrasound (POCUS) is a new modality which has demonstrated promise for the evaluation of appropriate TT positioning. For the perioperative setting, the principal investigator for this manuscript performed a randomized control trial in which adult patients were selected to have the TT placed either in the trachea or the R/L bronchi. This study compared the efficacy of detecting tracheal vs. bronchi TT position between stethoscope auscultation and a POCUS examination, termed PLUS (pulmonary tree and lung expansion ultrasound study). The results from this study demonstrate a significantly greater sensitivity and specificity in the PLUS exam vs. stethoscope auscultation [6]. This study was among the first to demonstrate the utility of POCUS for detecting the appropriate TT position in the adult perioperative setting.

In regard to children, the utility of POCUS for identifying the appropriate location of the TT has been demonstrated [10,11,12]. These examinations, however, vary depending on the child’s age, [11] often require lower-frequency probes [12], and may require non-traditional maneuvers to be effective [10]. Also, some POCUS examinations may not be useful for detecting TT positions that are at risk of malposition, since they do not allow for strategies for obtaining measurements of the TT within the trachea [13].

This study sought to evaluate a novel perioperative POCUS exam for pediatrics. The exam consisted of a modified PLUS exam (one additional ultrasound US view) that included the evaluation of the dilation of the trachea with TT cuff inflation along with the evaluation of bilateral pleural sliding. The theoretical benefit of this exam is that it may provide both the identification of a mainstem intubation (lack of pleural sliding) and a method of measuring the location of the TT within the trachea (localization of where cuff inflation is visualized). To evaluate the potential utility of this POCUS exam, TT location measurements obtained from a PLUS exam were compared with TT location measurements obtained from chest fluoroscopy over a wide age range of children under 10 years old. Our hypothesis was that a POCUS exam could be an alternative to chest fluoroscopy by demonstrating a strong degree of correlation in TT location measurements between the two modalities. Specifically, after adjusting for the subjects’ height and TT size, the authors evaluated the correlation between measurements from the cricoid cartilage to the TT cuff, obtained by POCUS, and measurements from the TT tip to the carina, obtained by fluoroscopy.

## 2. Methods

This article is presented following the Standards for the Reporting of Diagnostic Accuracy (STARD) checklist methodology for the reporting of studies of diagnostic accuracy.

### 2.1. Participants

The study was performed at Loma Linda University Medical Center after Institutional Review Board (IRB #5160372) approval from February 2017 to June 2018. Written informed consent and information release were obtained from parents or legal guardians prior to study participation. Assent was obtained from children aged 7 years or older who were able to read and understand a simplified description of the study. Patients scheduled for procedures in the cardiac catheterization laboratory were screened for this study. This patient population was selected for screening secondary to a current clinical protocol at the study institution for obtaining a chest fluoroscopy for the verification of appropriate TT placement prior to procedure initiation. Under this protocol, the fluoroscopic measurement of the TT tip’s distance from the carina was used to evaluate appropriate positioning. Additional inclusion criteria were children aged from birth to 10 years and general anesthesia with a cuffed TT. The exclusion criteria were emergency procedures, patients with known airway anomalies, and parents/guardians who were non-English- or non-Spanish-speaking.

Ultrasound training: A total of four physician examiners (two pediatric anesthesiology fellows, one anesthesiology resident, and one research fellow) performed the POCUS examinations. Each examiner received one-on-one instruction with the lead author of the original PLUS examination (D.R.) and each examiner performed 25 examinations prior to performing the study exams.

### 2.2. Protocol

**Figure 1:**
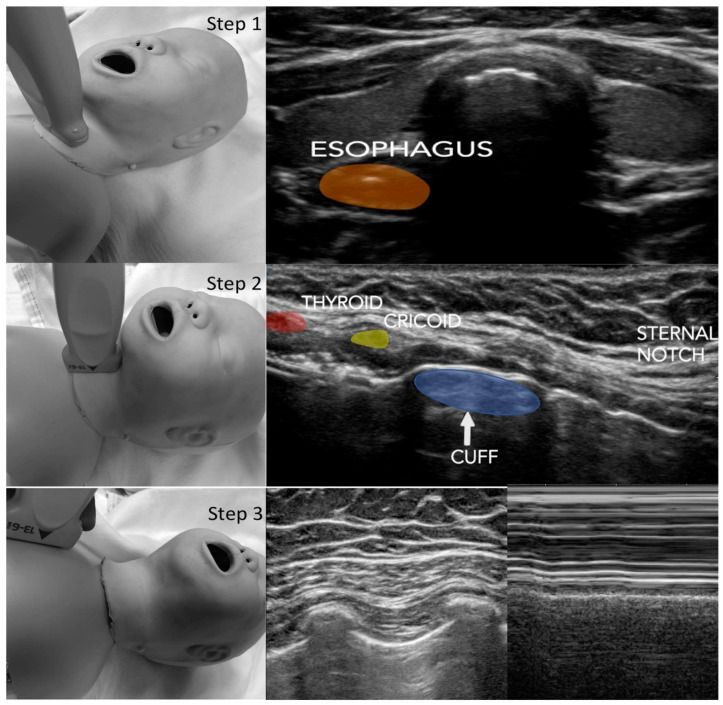
PLUS (pulmonary tree and lung expansion ultrasound study). Step 1: Trachea/Esophagus Assessment—The ultrasound probe is placed transversely on the anterior neck approximately 2 cm superior to the suprasternal notch and scanned cranially/caudad. Step 2: The probe is then rotated 90 degrees such that the long axis of the trachea is achieved with the cricoid cartilage. Step 3: Pleural Sliding Assessment—The ultrasound probe is placed vertically on the anterior chest at the third rib space midclavicular line bilaterally. The assessment of lung expansion is evaluated by the detection of the horizontal movement of the two pleural linings with respiration (lung sliding). The use of the M-mode facilitates the pleural sliding assessment.

All patients underwent tracheal intubation via direct laryngoscopy. The TT was positioned by the anesthesiologists using traditional methods including capnography verification, standard American Society of Anesthesiology recommended monitors, and stethoscope auscultation. After appropriate TT placement was confirmed via these techniques, the POCUS and fluoroscopy images were obtained sequentially. All patients received ShileyTM cuffed tracheal tubes from Covidien (Dublin, Ireland). The trained physician examiners performed the POCUS examination using an institutionally purchased Sonosite X-Porte (Fujifilm Sonosite, Bothell WA) ultrasound machine with a high-frequency linear probe (13–6 MHz). The examination consisted of the placement of the probe transversely on the anterior neck approximately 2 cm from the sternal notch. The probe was adjusted in the cephalad or caudad direction to identify the second tracheal ring and the esophagus location was then identified. The trachea was then placed in the middle of the ultrasound footprint and the probe was then rotated 90 degrees to achieve a long-axis cross-section of the airway anatomy (an additional view to those in the original PLUS examination). The probe was adjusted in either the cephalad or caudad direction such that the cricoid cartilage was identified on the end of the ultrasound image. The pilot balloon was inflated and deflated to identify the position of the TT cuff in the airway, as previously described [6]. The volume of air applied to the TT cuff was kept at the same amount administered during the primary TT placement. Additionally, manometry of the pilot balloon was used during this time to ensure that the cuff pressure was never greater than 20 cm H_2_O. The final component of the examination consisted of the placement of the probe on the left and right anterior chest walls, at the third rib space along the midclavicular line, to ascertain the presence of the pleural sliding sign. A graphic of the modified PLUS examination used for this study is shown in Figure 1. After the POCUS examination was complete, the patient immediately received an anterior-posterior (AP) chest fluoroscopy image per institutional protocol. Importantly, there was no change in patient position or adjustment of the TT location until both the POCUS and fluoroscopy images were obtained.

### 2.3. Data Acquisition

Patient demographics were captured from a review of the electronic medical record (Epic Systems Corporation, Verona, WI). Ultrasound measurements were performed immediately after the examination was performed. All ultrasound parameters, including image quality, distance measurements, identification of the esophagus, and the presence of lung sliding, were reviewed by the expert examiner (D.R.) who was blinded to patient identifiers and demographics. This examiner (D.R.) did not perform any of the study examinations, and rated image quality using a previously validated 1 (worst) to 5 (best) Likert scale [14]. Similarly, radiologic measurements were performed in intervals of ten-patient blocks by an anesthesiologist who was blinded to the ultrasound measurements.

Measurements of the TT location included a direct measurement obtained from both imaging modalities as well as the inclusion of scaled factors. The direct measurement from the ultrasound image was from the base of the cricoid cartilage to the center area identified during cuff inflation (Figure 1). The direct measurement from the fluoroscopy image was from the tip of the TT to the carina. Additional factors were included given the wide age range of patients included in this study. A weighted strategy was implemented to equalize each subject prior to analysis. Indeed, without a strategy to equalize the data, patients with smaller airway anatomy (younger in age) would have had a stronger influence. Thus, both POCUS and chest fluoroscopy CXR measurements were divided by the patient’s height to allow for a weighted correlation analysis. It is important to note that measurements using this scale are unit-less, since height, POCUS distance, and fluoroscopy distance are all measured in millimeters. Additionally, another variable that had to be addressed prior to analysis was the fact that the distance from the tip of the TT to the TT cuff changes based on the size of the TT. To prevent this from impacting the correlation analysis, the midline of the tracheal tube cuff to the tip of the TT was measured for all potential TT sizes (2.5 to 7 mm) and this value was added to the fluoroscopy measurement of the distance from the carina to the TT tip.

### 2.4. Statistical Analysis

Descriptive statistics were used for the following: baseline demographic data, time to perform the POCUS examination, and image quality categorization. The primary outcome marker for this study was the correlation between the weighted POCUS measurement of TT location and the weighted fluoroscopy measurement of TT location. The secondary outcome markers included a linear regression analysis of the scaled measurements obtained from the two imaging modalities with age included as a covariate. Additionally, the evaluation of associations between image quality and patient demographics was also reviewed. 

The Shapiro–Wilk test was used to evaluate for normality for all measurement variables. Regression analysis and Spearman’s rank correlation were used to compare the scaled POCUS measurements with the scaled fluoroscopy measurements as described above. The Breusch–Pagan test was used to check for heteroscedasticity in the regression models. Moreover, the R-squared value was presented to evaluate the degree of variance explained by the regression model with age included as a covariate. All statistical analyses were conducted in R version 3.4.0.

### 2.5. Sample Size

The effect size for this study was set as a correlation coefficient of 0.5, which has been referenced as a statistical standard for a moderate effect size [15]. Assuming the correlations between the POCUS scaled measurements and the fluoroscopy scaled measurements were to be applied to a bivariate Pearson’s correlation with α = 0.05 (two tailed test) and power = 0.90, a sample size of 37 was determined.

## 3. Results

Fifty patients consented to the study, with the final data analysis comparing 41 subjects. Of the nine subjects excluded from the study, four did not have their fluoroscopy images saved into the EMR system and five received a laryngeal mask airway instead of a TT. Subject demographics are shown in Table 1. The age range for the study was 8 weeks to 10 years. No patient had malposition of the TT after the initial placement during induction as verified by fluoroscopy. For all included patients, the carina was visualized by fluoroscopy. The median time to perform the POCUS exam was 112 s. All images were rated as 5/5 for image quality. Additionally, the POCUS exams demonstrated 100% localization of the esophagus, with 76% of the cases demonstrating the esophagus to be to the left of the trachea. Full details are listed in Table 1.

**Figure 2:**
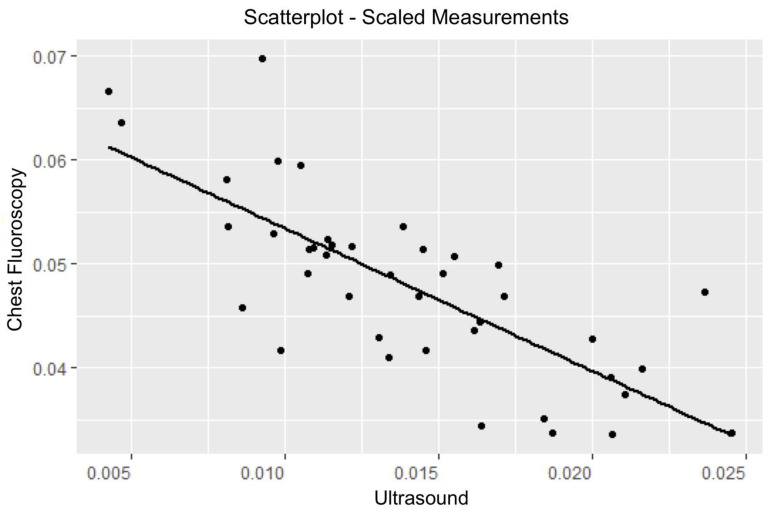
Scatter plot of scaled chest fluoroscopy vs. scaled ultrasound measurements.

Primary outcome analysis demonstrated significant correlations between the scaled POCUS and scaled fluoroscopy measurements, r = −0.7575, 95% CI [−0.8638, −0.5866], *p* < 0.001) (Figure 2). Secondary outcome analysis using a linear regression model between the scaled image modalities, with a covariate of subject age, demonstrated a significant relationship (R^2^ = 0.71, α = 0.05). Cross-validation was used to evaluate our linear regression model with *n* = 30 in the training set and *n* = 11 in the testing set. Moreover, we observed the values of 0.004588 and 0.005233 for the root mean square error of our training and testing sets, respectively; also, we observed the values of 0.003571 and 0.004669 for the mean absolute error for our training and testing sets, respectively. The residual normality requirement was not broken for the model and there was no heteroscedasticity present. Full details are listed in Table 2.

## 4. Discussion

This study further supports the utility of POCUS for evaluating TT position and providing a method of measuring the location of the TT within the trachea over a wide age range of pediatric patients. Specifically, our results demonstrate a statistically significant correlation between a proven technique of measuring TT localization (chest fluoroscopy) and a more novel technique (point-of-care ultrasound). Additionally, this study demonstrates that after receiving structured training, users can perform the described POCUS examination (PLUS exam) within 3 min, 85% percent of the time, yielding high image quality. Future studies could directly evaluate the performance of POCUS as compared to conventional radiologic methods as a primary means for measuring the location of the TT within the trachea.

The utility of POCUS for identifying the appropriate location of the TT in children has been demonstrated. In infants, the majority of these studies evaluated the distance between the tip of the TT, visualized with a curved low-frequency transducer, and the maximal curvature of the aortic arch [12]. While a proven effective technique, it is unlikely that this method will work in the older pediatric population. Additionally, the exam requires a low-frequency probe that is often not available in the perioperative setting. Regarding the pediatric population, a recent study demonstrated the utility of using POCUS to evaluate appropriate TT position by visualizing the cuff of the TT after saline inflation at the level of the sternal notch [10]. While this study showed very positive results, it is limited in its application in that saline cuff inflation (even if temporary) is not routinely done in most acute care settings. Importantly, this technique is similar to the PLUS exam with the exception that the PLUS exam evaluates the dilation of the trachea using cuff inflation with air. Another POCUS technique that has demonstrated utility in the pediatric population is the assessment of bilateral diaphragmatic motion (suggesting appropriate TT position) [11,13]. While this technique also demonstrates significant benefits, its limitations include the inability to measure the TT’s location within the airway. The inability to measure TT location prevents the detection of placements that are “at risk” of malposition. Ultimately, there are several methods of using POCUS in infants and children. This study demonstrates another effective strategy that may have widespread application and uses the most readily available ultrasound equipment in the perioperative setting.

However, despite being a growing area of interest, the use of point-of-care ultrasound is not mainstream in the perioperative environment. This was nicely summarized in a recent position paper by a consensus group of international experts in the fields of cardiothoracic, general, and critical care, pain management, and regional anesthesiology. This group reported a “call to action” on this topic and emphasized the importance for our specialty’s societies to develop standards of training such that proficiency in POCUS is expected for our specialty in the United States [16]. Further support for POCUS integration has also recently been supported in the United States by the Accreditation Council for Graduate Medical Education (ACGME), with 2018 program requirements for anesthesiology requiring “competency in using surface ultrasound and transthoracic echocardiography to guide the performance of invasive procedures and to evaluate organ function and pathology as related to anesthesia, critical care and resuscitation” [17]. This project further supports these initiatives by demonstrating the utility of perioperative airway ultrasound for the localization of the TT in children.

This study has several limitations. First, it is important to acknowledge that this study was an observational study designed to assess the efficacy of TT location measurements obtained from a novel POCUS examination for children. The goal of this study was to evaluate whether POCUS could be used as another tool to measure the location of the TT, which has the primary benefit of not requiring radiation exposure. This concept should be applied to scenarios in which TT location measurements are useful. There was no component of this study that evaluated the utility of applying POCUS routinely for intubations. Secondly, all patients in this study demonstrated the appropriate positioning of their TT with primary placement, and so no direct evaluation of inappropriate TT placement was able to be performed. It is the authors’ hope that future studies can evaluate the utility of POCUS in this manner. Thirdly, as with most diagnostic tools, there is a possibility for error in the ability to both perform and interpret the exam. While this study demonstrated a high image quality for all POCUS examinations, only a few providers performed the ultrasound exams. Further studies are needed to evaluate the ability to train a larger group of providers in the PLUS exam. Also, the TTs used during this study were all from the same manufacturer; however, the authors feel that this should minimally impact the ultrasound and fluoroscopy measurements. Finally, the small sample size, wide age range of children enrolled, and inclusion of patients only undergoing cardiac catherization procedures are additional limitations. For this reason, we did not separate our analysis by any subgroup, such as age or gender. Indeed, the goal of this study was to demonstrate the feasibility of applying a single POCUS examination across a wide age range of children. Future studies with larger sample sizes are needed to evaluate the utility of applying the PLUS exam in these subgroups.

## 5. Conclusions

This study supports the utility of using ultrasound as an accurate method of measuring the location of the tracheal tube within the trachea in a wide age range of children. Future studies could directly evaluate the performance of POCUS as compared to conventional radiologic methods as a primary means for measuring the location of the TT within the trachea.

## Figures and Tables

**Table 1:** Study characteristics: chest fluoroscopy to ultrasound.

Characteristics		
*N*	Count	41
Age (in years)	Median (25th Percentile, 75th Percentile)	3.00 (1.50, 7.00)
0–2 years	Count (Percentage)	18 (44%)
3–6 years	Count (Percentage)	11 (27%)
7–10 years	Count (Percentage)	12 (29%)
Sex (M:F)	Count (Percentage)	21 (51%):20 (49%)
Weight (in kilograms)	Median (25th Percentile, 75th Percentile)	14.30 (9.20, 22.60)
Height (in centimeters)	Median (25th Percentile, 75th Percentile)	96.00 (79.50, 123.00)
ASA (2:3:4)	Count (Percentage)	17 (42%):23 (56%):1 (2%)
Location of Esophagus at Second Tracheal Ring via POCUS Exam Left: Right: Middle	Count (Percentage)	31 (76%):8 (20%):2 (5%)
POCUS Image Quality Scores (1—Low to 5—High)	Count (Percentage)	1 (0%):2 (0%):3 (0%):4 (0%):5 (100%)
POCUS Examination Time (in seconds)	Median (25th Percentile, 75th Percentile)	112.00 (80.00, 156.00)

**Table 2:** Linear regression: chest fluoroscopy measurements vs. ultrasound measurements.

Coefficients	Estimates	Standard Error	*p*-Values
Intercept	0.07086	0.002903	<0.0001
Ultrasound	−1.4231	0.2057	<0.0001
Age	−0.0008130	0.0002909	0.008130
	R-Squared of Model	0.7094	

## Implication Statement

Point-of-care airway ultrasound has demonstrated utility in the pediatric population but there is limited information on its ability to be measure the location of the tracheal tube cuff within the trachea. This study demonstrates a novel point-of-care airway ultrasound examination that offers a high degree of image quality over a wide age range and provides measurements of tracheal tube cuff location with a strong correlation with routine fluoroscopy measurements.

## References

[1] Miller A., Mandeville J. (2016). Predicting and measuring fluid responsiveness with echocardiography. Echo. Res. Pract..

[2] Freeman J.A., Fredricks B.J., Best C.J. (1995). Evaluation of a new method for determining tracheal tube length in children. Anaesthesia.

[3] Loew A., Thibeault D.W. (1974). A new and safe method to control the depth of endotracheal intubation in neonates. Pediatrics.

[4] Harris E.A., Arheart K.L., Penning D.H. (2008). Endotracheal tube malposition within the pediatric population: A common event despite clinical evidence of correct placement. Can. J. Anaesth..

[5] Verghese S.T., Hannallah R.S., Slack M.C., Cross R.R., Patel K.M. (2004). Auscultation of bilateral breath sounds does not rule out endobronchial intubation in children. Anesth. Analg..

[6] Ramsingh D., Frank E., Haughton R., Schilling J., Gimenez K.M., Banh E., Rinehart J., Cannesson M. (2016). Auscultation versus Point-of-care Ultrasound to Determine Endotracheal versus Bronchial Intubation: A Diagnostic Accuracy Study. Anesth..

[7] Koshy T., Misra S., Chatterjee N., Dharan B.S. (2016). Accuracy of a Chest X-Ray-Based Method for Predicting the Depth of Insertion of Endotracheal Tubes in Pediatric Patients Undergoing Cardiac Surgery. J. Cardiothorac. Vasc. Anesth..

[8] Gamble J.J., McKay W.P., Wang A.F., Yip K.A., O’Brien J.M., Plewes C.E. (2014). Three-finger tracheal palpation to guide endotracheal tube depth in children. Paediatr. Anaesth..

[9] Sugiyama K., Yokoyama K. (1996). Displacement of the endotracheal tube caused by change of head position in pediatric anesthesia: Evaluation by fiberoptic bronchoscopy. Anesth. Analg..

[10] Tessaro M.O., Salant E.P., Arroyo A.C., Haines L.E., Dickman E. (2015). Tracheal rapid ultrasound saline test (T.R.U.S.T.) for confirming correct endotracheal tube depth in children. Resuscitation.

[11] Jaeel P., Sheth M., Nguyen J. (2017). Ultrasonography for endotracheal tube position in infants and children. Eur. J. Pediatr..

[12] Chowdhry R., Dangman B., Pinheiro J.M. (2015). The concordance of ultrasound technique versus X-ray to confirm endotracheal tube position in neonates. J. Perinatol..

[13] Kerrey B.T., Geis G.L., Quinn A.M., Hornung R.W., Ruddy R.M. (2009). A prospective comparison of diaphragmatic ultrasound and chest radiography to determine endotracheal tube position in a pediatric emergency department. Pediatrics.

[14] Levine A.R., McCurdy M.T., Zubrow M.T., Papali A., Mallemat H.A., Verceles A.C. (2015). Tele-intensivists can instruct non-physicians to acquire high-quality ultrasound images. J. Crit. Care.

[15] Sullivan G.M., Feinn R. (2012). Using Effect Size-or Why the P Value Is Not Enough. J. Grad. Med. Educ..

[16] Mahmood F., Matyal R., Skubas N., Montealegre-Gallegos M., Swaminathan M., Denault A., Sniecinski R., Mitchell J.D., Taylor M., Haskins S. (2016). Perioperative Ultrasound Training in Anesthesiology: A Call to Action. Anesth. Analg..

[17] ACGME Program Requirements for Graduate Medical Education in Anesthesiology. https://www.acgme.org/Portals/0/PFAssets/ProgramRequirements/040Anesthesiology2018TCC.pdf?ver=2018-06-14-143123-497.

